# Chidamide, decitabine, cytarabine, aclarubicin, and granulocyte colony-stimulating factor (CDCAG) in patients with relapsed/refractory acute myeloid leukemia: a single-arm, phase 1/2 study

**DOI:** 10.1186/s13148-020-00923-4

**Published:** 2020-09-01

**Authors:** Lixin Wang, Jianmin Luo, Guofeng Chen, Meiyun Fang, Xudong Wei, Yinghua Li, Zhuogang Liu, Yin Zhang, Sujun Gao, Jianliang Shen, Xin Wang, Xiaoning Gao, Wei Zhou, Yigai Ma, Hui Liu, Xinquan Li, Linhua Yang, Kai Sun, Li Yu

**Affiliations:** 1 Department of Hematology-Oncology, International Cancer Center, Shenzhen University General Hospital, Shenzhen University Health Science Center, Shenzhen, China; 2 Department of Hematology, The Sixth Medical Center, Chinese General Hospital of PLA, Beijing, China; 3 Department of Hematology, The Second Hospital of Hebei Medical University, Shijiazhuang, China; 4 Department of Endoscopy, Tianjin Medical University Cancer Institute and Hospital, National Clinical Research Center for Cancer, Tianjin’s Clinical Research Center for Cancer, Key Laboratory of Cancer Prevention and Therapy, Tianjin, China; 5 School of Medicine, Nankai University, Tianjin, China; 6 Department of Hematology, Chinese PLA General Hospital, 28 Fuxing Road, Beijing, 100853 China; 7 Department of Hematology, The First Affiliated Hospital of Dalian Medical University, Dalian, China; 8 Department of Hematology, The Affiliated Cancer Hospital of Zhengzhou University, Henan Cancer Hospital, Zhengzhou, China; 9 Department of Hematology, The First Affiliated Hospital of Harbin Medical University, Harbin, China; 10 Department of Hematology, Shengjing Hospital of China Medical University, Shenyang, China; 11 Department of Hematology, Henan Provincial People’s Hospital, Zhengzhou, China; 12 Department of Hematology, The First Hospital, Jilin University, Changchun, China; 13 Department of Hematology, Shandong Provincial Hospital Affiliated to Shandong University, Jinan, China; 14 Department of Hematology, China­Japan Friendship Hospital, Beijing, China; 15 Department of Hematology, Beijing Hospital, National Center of Gerontology, Beijing, China; 16 Department of Hematology, Beijing Tsinghua Changgung Hospital, Tsinghua University, Beijing, China; 17 Department of Hematology, The Second Hospital of Shanxi Medical University, Taiyuan, China

**Keywords:** Relapsed/refractory acute myeloid leukemia, Next-generation sequencing, Histone deacetylase inhibitor, DNA methyltransferase inhibitor

## Abstract

**Background:**

Epigenetic mechanisms play an important role in the chemoresistance of acute myeloid leukemia (AML). The clinical response to epigenetic modifier-based chemotherapy in patients with relapsed/refractory AML (r/r AML) is unclear. This multicenter clinical trial evaluated the safety and efficacy of epigenetic modifiers (chidamide and decitabine) in combination with aclarubicin, cytarabine, and granulocyte colony-stimulating factor (G-CSF) in patients with r/r AML.

**Results:**

Adult patients with r/r AML were treated with chidamide, decitabine, cytarabine, aclarubicin, and G-CSF (CDCAG). The primary measures were overall response (OR), overall survival (OS), and safety. Next-generation sequencing was performed to analyze the correlation between gene mutations and response. A total of 93 patients with r/r AML were enrolled. Overall, 24 patients had a complete remission (CR) and 19 patients achieved CR with incomplete blood count recovery (CRi). The overall response rate (ORR) was 46.2%. The overall survival of these 43 patients who achieved CR/CRi was significantly longer than that of patients who failed to achieve remission (563 vs 152 days, *P* < 0.0001). Of the patients with mutations in epigenetic and transcription factor-related genes, but without internal tandem duplications in FMS-like tyrosine kinase3 (*FLT3*-ITDs), 55.6% achieved CR/CRi, whereas the ORR was 28.2% for patients with mutations in other genes.

**Conclusions:**

The CDCAG regimen was well tolerated and effective in r/r AML. Patients with epigenetic and transcription factor-related gene mutations, but without *FLT3*-ITD mutations, may benefit from this regimen.

**Trial registration:**

Clinical Trials, NCT02886559. Registered 01 September 2016

## Introduction

Approximately 30% of acute myeloid leukemia (AML) cases will be classified as refractory AML due to failure of induction chemotherapy. Additionally, more than 50% of patients who achieve complete remission (CR) will eventually relapse [1]. Therefore, the majority of patients with AML will eventually be classified as refractory or relapsed AML (r/r AML). The prognosis for r/r AML remains dismal despite significant effort devoted to the development of novel single-agent drugs and the design of new combination regimens.

Resistance to multiple chemotherapeutic agents is a common clinical problem encountered in r/r AML treatment [2]. Accumulating research has demonstrated the importance of epigenetic modification in the pathogenesis of chemoresistance. DNA methylation and histone acetylation are the most common epigenetic changes and can be pharmacologically reversed by DNA methyltransferase (DNMT) inhibitors or histone deacetylase (HDAC) inhibitors. Recent studies have shown that decitabine, a DNMT inhibitor, can increase the chemosensitivity of several leukemic [3] and solid tumor cells [4, 5]. Furthermore, the addition of HDAC inhibitors, such as chidamide or panobinostat, can enhance decitabine’s chemosensitization and cytotoxicity effects on leukemia cells when combined with conventional chemotherapy [6–9].

The standard dose of CAG regimen, consisting of low-dose cytarabine, aclarubicin, and granulocyte colony-stimulating factor (G-CSF), led to a CR rate of 50% with well-tolerated toxicity in elderly patients, and the combination with decitabine increased the CR rate to 64.7% [10–12]. In this regimen, aclarubicin, an anthracycline topoisomerase II inhibitor, was recently reported to induce histone eviction in genomic regions and caused important epigenetic changes [13, 14]. Therefore, exploring whether the addition of epigenetic modifiers, such as decitabine and further the HDAC inhibitors, to the CAG regimen could exert a clinical benefit in r/r AML patients is of great significance. In this study, we designed a regimen that included chidamide, decitabine, cytarabine, aclarubicin, and G-CSF (the CDCAG regimen) for the treatment of patients with r/r AML. We then conducted a phase I/II study to evaluate the safety and efficacy of this regimen.

## Results

### Patient characteristics

A total of 93 patients, median age 40 years (range, 18–60 years), with primary refractory AML (*n* = 37, 39.8%), early relapsed AML (*n* = 38, 40.9%), or late relapsed AML (*n* = 18, 19.4%) were enrolled in the study. Among the 56 patients with relapsed AML, 40 were experiencing their first relapse, 15 were in their second relapse, and one was experiencing a third relapse. Patients with relapsed AML had a median remission duration before relapse of 7.6 months (range, 1–51 months). Sixty-nine (74.2%) patients had received at least three prior treatment regimens, 52 (55.9%) patients had received at least five prior treatment regimens, and 36 (38.7%) patients had received at least seven prior treatment regimens. Seventeen (18.3%) patients had received at least one hypomethylating agent, and two patients (2.2%) had previously undergone allogeneic hematopoietic stem cell transplantation (allo-HSCT). Patients had received a median of five (1–17) therapeutic cycles prior to enrollment in this study. Patients were enrolled at a median of 8.8 months (1.3–63.8 months) after initial diagnosis. Patient baseline characteristics are summarized in Table 1.

**Table 1:** Patient demographics and baseline characteristics (*N* = 93)

Characteristic	Value^$^
Age, years	40.0 ± 12.4
Sex, no. (%)	
Male	50 (53.8)
Female	43 (46.2)
BM blasts, %	0.4 ± 0.3
HB, g/dL	91.0 ± 25.3
WBC, × 10^9^/L	9.2 ± 15.9
PLT, × 10^9^/L	69.5 ± 66.4
ECOG PS, no. (%)	
0	34 (36.6)
1	42 (45.2)
2	16 (17.2)
3	1 (1.1)
FAB classification, no. (%)	
M0	2 (2.2)
M1	3 (3.2)
M2	49 (52.7)
M4	14 (15.1)
M5	24 (25.8)
M6	1 (1.1)
Diagnosis, no. (%)	
Refractory	37 (39.8)
Early relapse	38 (40.9)
Late relapse	18 (19.4)
Antecedent hematologic disorders, no. (%)	
Myelodysplastic syndromes	3 (3.2)
Aplastic anemia	1 (1.1)
Prior therapies, no. (%)	
0–5	52 (55.9)
6–10	28 (30.1)
≥ 11	13 (14.0)
Prior therapy, no. (%)	
Prior epigenetic agents	17 (18.3)
Prior allogeneic stem cell transplant	2 (2.2)
Karyotype, no. (%)	
Normal karyotype	54 (58.1)
Complex karyotype^#^	9 (9.7)
T (8; 21)	13 (14.0)
Mutation counts, no. (%)*	
0	12 (13.6)
1	20 (22.7)
2	22 (25.0)
3	19 (21.6)
4	12 (13.6)
5	3 (3.4)

*Abbreviations*: *BM* bone marrow, *HB* hemoglobin, *WBC* white blood cell count, *PLT* platelets, *ECOG PS* Eastern Cooperative Oncology Group performance score, *FAB* French-American-British, *CEBPA-dm* CEPBA double mutation

^$^Descriptive statistics were presented as the mean ± standard deviation (mean ± SD) for continuous data and as numbers and percentages for dichotomous/categorical data

*Measured in 88 patients who underwent gene mutation detection

^#^Complex karyotype was defined as ≥ 3 clonal chromosomal abnormalities

### Clinical responses

Overall, 24/93 (25.8%) patients achieved CR according to the International Working Group (IWG) criteria and an additional 19/93 (20.4%) patients achieved CR with incomplete blood count recovery (CRi), with an overall response rate (ORR) of 46.2% (Table 2). Among the 43 patients who achieved CR/CRi, the median duration of leukemia-free survival was 259 days [95% confidence interval (CI) 215—not available]. Of the 17 patients who received prior hypomethylating agents, nine (52.9%) achieved CR/CRi.

**Table 2:** Best response after 1–2 cycles of the CDCAG therapy (*N* = 93)

Clinical response	*N* (%)
CR	24 (25.8)
CRi	19 (20.4)
PR	8 (8.6)
NR	38 (40.9)
ED	4 (4.3)

*Abbreviations*: *CR* complete remission, *CRi* complete remission with incomplete blood count recovery, *PR* partial remission, *NR* no response, *ED* early death

**Fig. 1:**
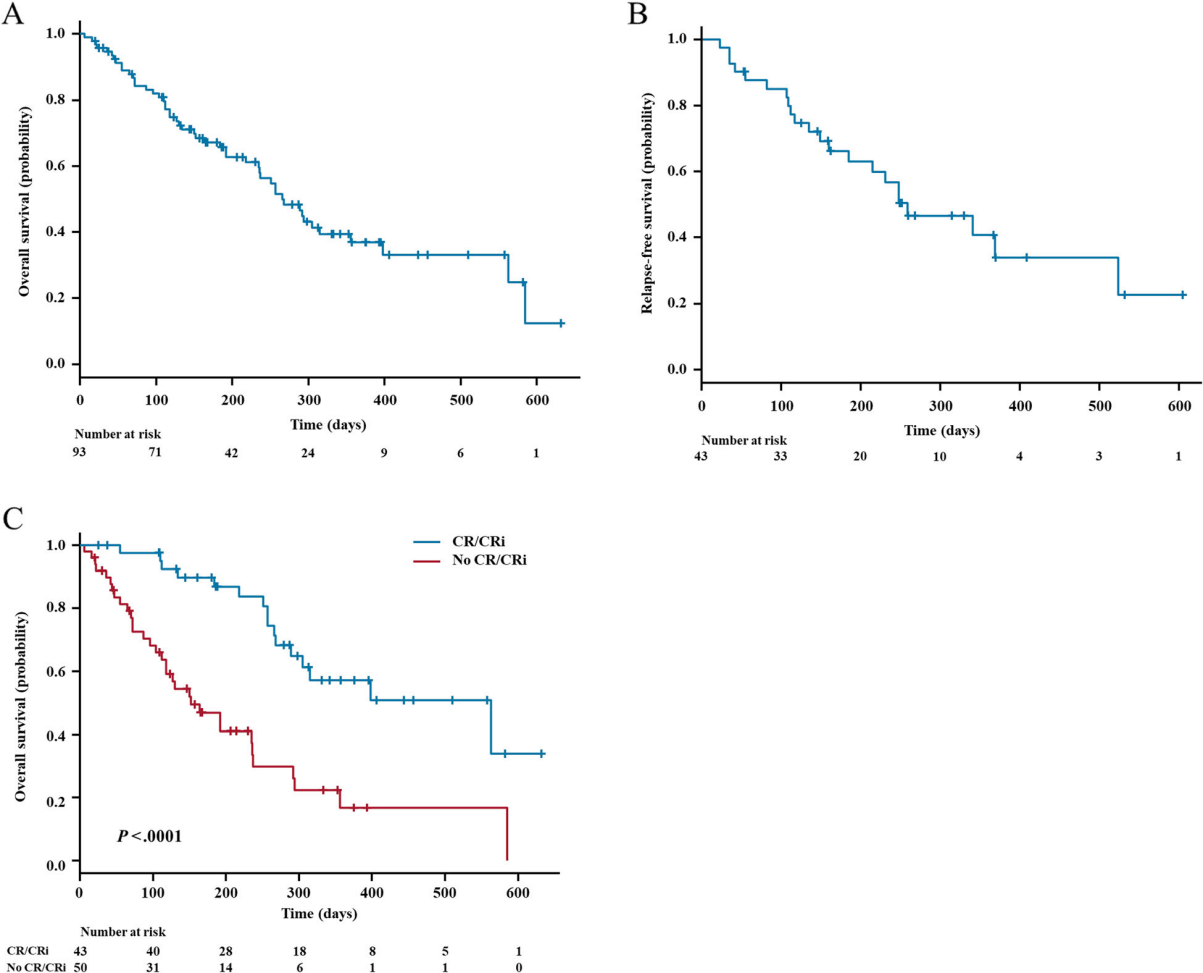
Survival curves. **a** Overall survival curves for 93 patients with r/r AML. **b** Relapse-free survival curves for 43 patients with r/r AML. **c** Overall survival curves for 93 patients with r/r AML. Data are categorized according to whether CR/CRi was achieved. r/r AML, relapse/refractory acute myeloid leukemia; CR/CRi, complete remission or complete remission with incomplete count recovery

The median overall survival (OS) was 266 days (95% CI 235–398 days) with an estimated 6-month OS rate of 67.2% (95% CI 57.8–78.0%) and a 1-year OS of 36.9% (95% CI 26.6–51.1%, Fig. 1a). For the 43 patients who achieved CR/CRi, the 6-month relapse-free survival (RFS) rate was 66.2% (95% CI 52.7–83.2%), the 1-year RFS rate was 40.7% (95% CI 26.0–63.9%), and the median RFS was 259 days (95% CI 185–NA; Fig. 1b). Patients achieving CR/CRi showed a significant improvement in median OS compared with that of non-responders (563 vs 152 days, *P* < 0.0001; Fig. 1c). However, the differences of ORR and OS among patients with primary refractory AML, those in their first relapse, and those who had experienced multiple relapses were not statistically significant (data not shown).

Of the 43 patients who achieved CR/CRi, eight patients underwent allo-HSCT treatment. Among these eight patients, the death rate was 12.5%, whereas for the remaining 35 patients who received other treatments, the death rate was 42.9%. Moreover, patients who underwent allo-HSCT showed a trend of longer OS than those received other treatments, although it was not statistically significant (*P* = 0.0546; Fig. S1).

### Molecular features and patient characteristics

**Fig. 2:**
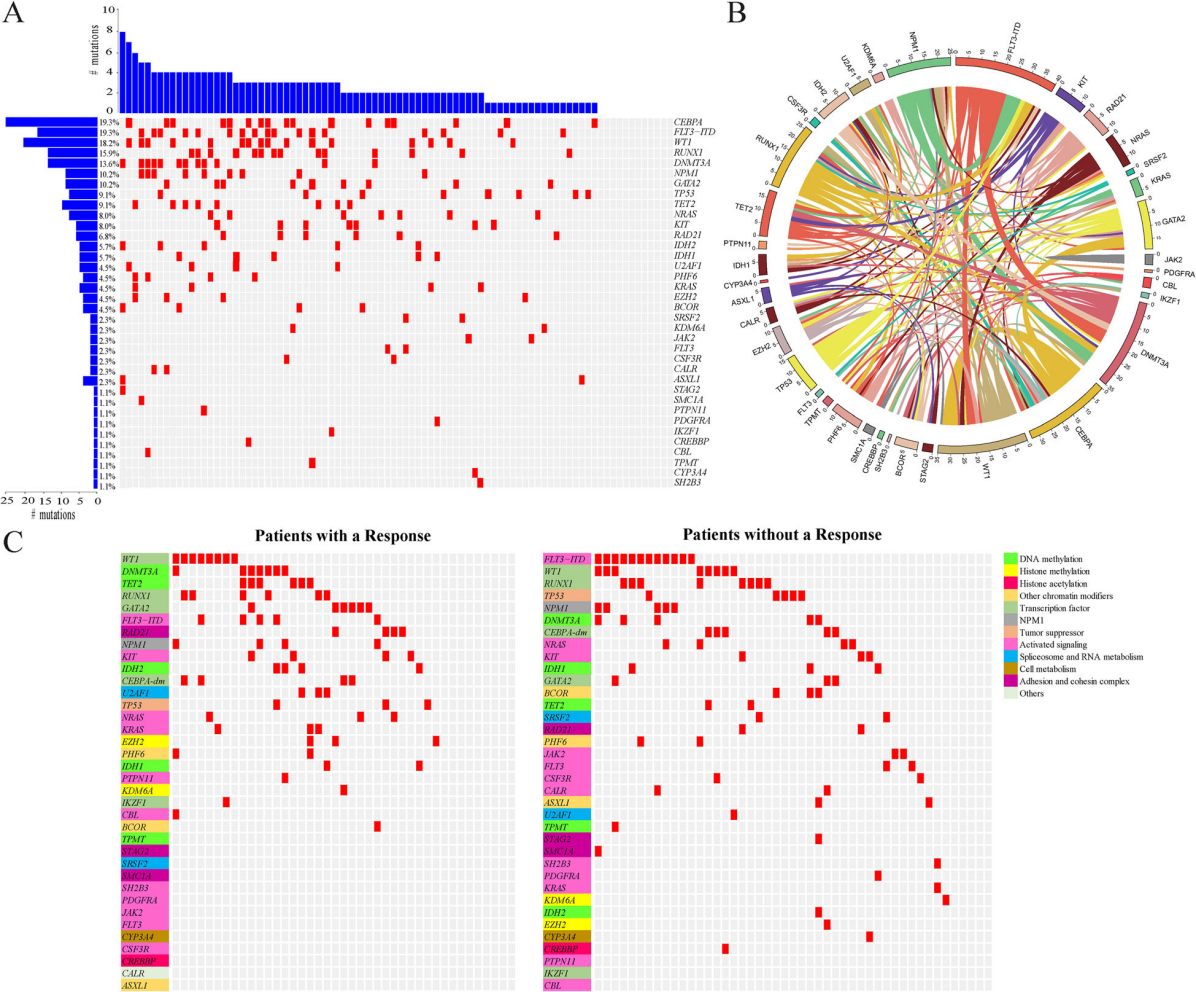
Correlation between somatic mutations and clinical responses. **a** Landscape of mutations detected in 88 patients at enrollment. Each row represents a gene, and each column corresponds to a participant in the study. The number of patients with mutations is listed on the left. Bar plots indicate the number of mutations per patient (top bar plot) and the number of mutations detected for each gene (side bar plot). **b** Co-mutations among the 88 patients with gene mutation detection. The thickness of the connecting lines indicates the frequency with which the two mutations co-occurred. **c** Landscape of mutations detected in 88 patients at enrollment, according to whether the patient achieved a response (complete remission or complete remission with incomplete count recovery) or not. Each row represents a gene, and each column corresponds to a participant in the study. CEBPA-dm, CEBPA double mutation

In total, 88/93 patients underwent gene mutation detection with next-generation sequencing (NGS). Two hundred nine mutations in 184 genes were detected in 76 patients with a median of two mutated genes per patient (range, 0–5). There were 10 mutations found in one patient, seven mutations found in two patients, five mutations in four patients, two mutations in five patients, and 12 mutations in more than five patients. As shown in Fig. 2a and Table S1, the most frequently mutated genes were *FLT3*-ITD (19.3%), followed by *CEBPA* (19.3%, including 9.7% with a *CEBPA*-double mutation [*CEBPA*-dm]), *WT1* (18.2%), *RUNX1* (15.9%), and *DNMT3A* (13.6%). No significant differences in the number of mutated genes and their variant allele fractions (VAFs) were found among those experiencing refractory AML, first AML relapse, and second or more relapse AML (data not shown).

Among 76 patients with detectable mutations, 20 patients had one mutated gene, 22 patients had two mutated genes, 19 patients had three mutated genes, 11 patients had four mutated genes, and four patients had five mutated genes. In total, 73.7% (56/76) of the patients harbored co-mutations. The most common co-mutations were CEBPA with *WT1*, *NPM1* with *FLT3*-ITD, *DNMT3A* with *FLT3*-ITD, and *DNMT3A* with *NPM1* (Fig 2b). Among the 88 patients who underwent gene mutation detection, 12 patients had no detectable mutation. No significant differences in age, sex, bone marrow (BM) blasts, white blood cell (WBC), and karyotype were found between the patients with detectable mutations and those without (Table S2).

### Correlations between mutation profiles and responses

Among the 76 patients who had detectable gene mutations, 33 (43.4%) showed a response (CR/CRi), while 8/12 (66.7%) without detectable mutation showed a response. Patients with mutations in *IDH2*, *TET2*, *GATA2*, *RAD21*, and *DNMT3A* had ORRs of up to 80.0%, 75.0%, 66.7%, 66.7%, and 58.3%, respectively, while patients with *FLT3*-ITD had an ORR of 29.4% (Table S3 and Fig. S2). However, none of the mutated genes, as well as the co-mutations, were statistically associated with a better ORR in univariate models (Table S4 and Fig. S3).

**Fig. 3:**
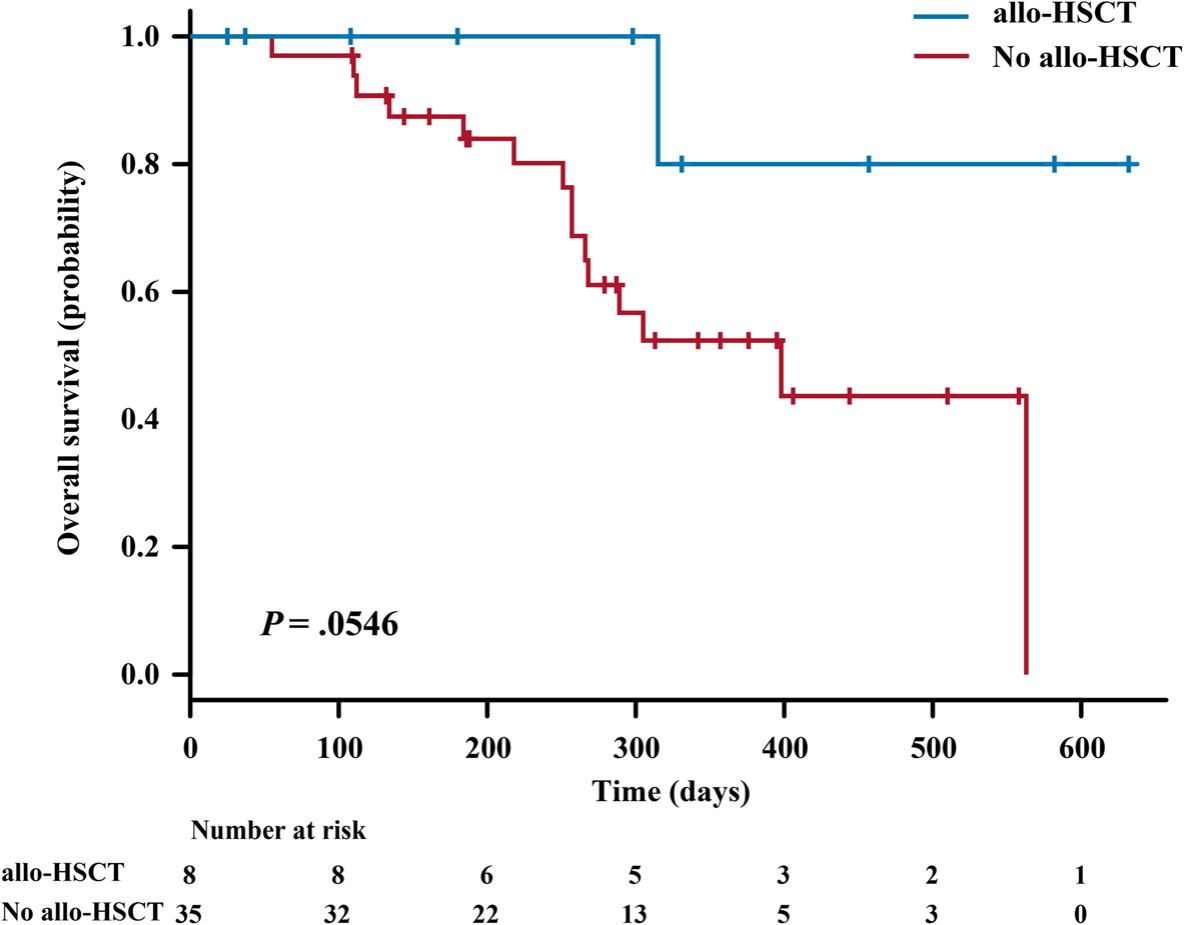
Overall survival curves for 76 patients with detectable mutation. Data are categorized according to whether mutation was in panel ET*. *Panel ET: epigenetic modifier-related or transcription factor-related gene mutations, but without FLT3-ITD co-mutation

Interestingly, the mutation profile differed between patients with a response and those without. To further analyze the correlation between gene mutation and clinical response, we categorized the genes into different functional groups, including DNA methylation-related gene mutations (found in 6.8% of patients), histone methylation-related gene mutations (4.5%), histone acetylation-related gene mutations (2.3%), transcription factor-related gene mutations (18.2%), and activated signaling-related gene mutations (44.3%) [15–17] (Table S5). As shown in Fig. 2c, patients who achieved CR/CRi showed more epigenetic modifier-related or transcription factor-related gene mutations but less FLT3-ITD mutations. We then defined the panel, including epigenetic modifier-related or transcription factor-related genes, but without FLT3-ITD co-mutation, as panel ET. The baseline characteristics were comparable between patients in the panel ET group and those with other mutations (Table S6). We observed that 22/37 (59.5%) patients in the panel ET group and 11/39 (28.2%) patients with other mutations achieved CR/CRi (*P* = 0.006), and the difference in ORR was also significant in the multivariate analyses (odds ratio = 4.45, *P* = 0.0085; Table 3). Compared with patients with other mutations, patients in the panel ET group had a better OS (*P* = 0.0460; Fig. 3). Moreover, when we excluded the FLT3-ITD mutation, patients in the panel ET group still had a better ORR (59.5% vs 27.3%, *P* = 0.0170) than those with other mutations, although there was no difference in OS (*P* = 0.13).

**Table 3:** Univariate and multivariate models of ORR for patients categorized according to their mutations (*N* = 76 (measured in 76 patients who had detectable gene mutations))

	Univariate models		Multivariate models	
	OR (95% Cl)	*P* value	OR (95% Cl)	*P* value
Category				
Other mutations	1.0		1.0	
Mutations in panel ET^#^	3.73 (1.43, 9.73)	0.0070	4.45 (1.46, 13.50)	0.0085
Age	1.03 (0.99, 1.07)	0.0920	1.04 (0.99, 1.08)	0.1351
Sex				
Male	1.0		1.0	
Female	0.43 (0.17, 1.11)	0.0823	0.39 (0.13, 1.21)	0.1035
BM blasts	0.13 (0.02, 0.80)	0.0275	0.32 (0.04, 3.01)	0.3218
HB	1.02 (1.00, 1.04)	0.0244	1.01 (0.99, 1.04)	0.2979
WBC	1.00 (0.98, 1.03)	0.9117		
PLT	1.01 (1.00, 1.01)	0.1566		
ECOG PS				
0	1.0			
1	0.24 (0.08, 0.78)	0.0169		
2	0.73 (0.19, 2.90)	0.6580		
3	0.00 (0.00, Inf)	0.9913		
Diagnosis				
Refractory	1.0		1.0	
Early relapse	2.00 (0.69, 5.78)	0.2002	1.81 (0.51, 6.47)	0.3600
Late relapse	4.27 (1.09, 16.83)	0.0377	4.91 (0.90, 26.80)	0.0663
Prior therapies				
0–5	1.0			
6–10	1.32 (0.48, 3.66)	0.5912		
≥ 11	1.87 (0.49, 7.18)	0.3588		

*Abbreviations*: *OR* odds ratio, *CI* confidence interval, *BM* bone marrow, *HB* hemoglobin, *WBC* white blood cell count, *PLT* platelets, *ECOG PS* Eastern Cooperative Oncology Group performance score

^#^Panel ET: epigenetic modifier-related or transcription factor-related gene mutations, but without FLT3-ITD co-mutation

### Safety

The CDCAG regimen was generally well tolerated in patients with r/r AML. Adverse events (AEs) were reported for all patients and are summarized in Table 4. The most common non-hematologic AEs were infection (including pneumonia), nausea, fatigue, vomiting, hypokalemia, hypoalbuminemia, and febrile neutropenia, the majority of which were grade 1/2. The most common grade 3/4 AEs were infection (including pneumonia) and febrile neutropenia. Serious AEs occurred in 12 (13%) patients, primarily due to sepsis (*n* = 3), pneumonia (*n* = 3), and cerebral hemorrhage (*n* = 2). There were no instances of tumor lysis syndrome. In addition, four (4.3%) patients experienced early death (death within 28 days of the start of treatment) due to cerebral hemorrhage (*n* = 2) and sepsis (*n* = 2); only one patient died of disease progression during consolidation.

**Table 4:** Treatment-related non-hematologic adverse events

AE*	Any grade AE, *N* (%)	Grade 3/4 AE, *N* (%)
Any AE	93 (100.0)	59 (63.4)
Infection**	43 (46.2)	25 (26.9)
Nausea	41 (44.1)	0 (0.0)
Fatigue	31 (33.3)	1 (1.1)
Vomiting	31 (33.3)	1 (1.1)
Hypokalemia	29 (31.2)	9 (9.7)
Hypoalbuminemia	25 (26.9)	0 (0.0)
Febrile neutropenia	23 (24.7)	23 (24.8)
Pneumonia	21 (22.6)	13 (14.0)
Hypocalcemia	18 (19.4)	1 (1.1)
Cough	17 (18.3)	0 (0.0)
Hyponatremia	16 (17.2)	2 (2.2)
Abdominal pain	14 (15.1)	0 (0.0)
Pharyngalgia	14 (15.1)	1 (1.1)
Anorexia	12 (12.9)	2 (2.2)
Diarrhea	10 (10.8)	0 (0.0)

*Abbreviations*: *AE* adverse event

*AEs were assessed based on the CTCAE (NCI Common Terminology Criteria for Adverse Events) version 5.0 and are shown with a frequency ≥ 10%

**Infection included pneumonia

## Discussion

Globally, the outcomes of patients with r/r AML are poor. The chance of achieving CR with commonly used salvage regimens, including high-dose cytarabine, is less than 15%, and the 1-year OS rate is less than 10% [18, 19]. In this prospective multicenter trial, 39.8% of the patients were diagnosed with primary refractory AML, and 67.9% of patients with relapsed disease were classified as early relapsed AML. The patients were treated with the CDCAG regimen. The CR/CRi rate was 46.2% and the median OS was 266 days, with a 1-year OS rate of 36.9%. Thus, the clinical results are encouraging, although it is difficult to compare our study to other trials directly because of the heterogeneity in the population. Notably, NGS was performed on 88 patients in order to explore the prognosis-associated gene mutation profile. Patients with epigenetic or transcription factor-related gene mutations, but without *FLT3*-ITD mutations, achieved better responses, which indicated that patients with r/r AML may benefit from our regimen.

Chemoresistance is the greatest challenge to be conquered for r/r AML patients. In this study, 69 (74.2%) patients had received at least three cycles of chemotherapy before enrollment, suggesting that leukemic cells in most patients were chemoresistant. In a recent study, cytosine methylation sequencing of genetically diverse AML patients revealed that many of the common AML driver mutations were epigenetic modifiers [20]. Furthermore, a direct epigenetic mechanism for AML chemoresistance was discovered by another study [21]. These studies suggest that the targeting of epigenetic modifiers may represent a new approach to overcome the chemoresistance of r/r AML cells. Decitabine is the first DNMT inhibiter that was used as an induction or salvage therapy to manage AML patients [22–24]. As a single agent, decitabine treatment achieved a CR rate of 15–21% in patients with r/r AML [24, 25]. Preclinical studies have shown that hypomethylating agents exhibit synergistic activity in leukemia cells when combined with an HDAC inhibitor [9, 26, 27]. Several clinical trials have explored the clinical benefit of a DNMT inhibitor in combination with an HDAC inhibitor for AML patients [28, 29]. The results of these studies were unsatisfactory since the CR/CRi rate was lower than 15%. In one study, decitabine was given for priming before cytotoxic agents mitoxantrone, etoposide, and cytarabine [30]. While 33% of patients with r/r AML achieved CR/CRi, seven patients (15%) died within 28 days of treatment initiation. In our trial, two classic epigenetic modifiers were included in the regimen: decitabine and chidamide. Chidamide is reported to act synergistically with DNA-damaging agents (daunorubicin, idarubicin, and cytarabine) or decitabine to diminish tumor burden in patients with r/r AML [6, 31]. Recently, aclarubicin, an anthracycline topoisomerase II inhibitor, was shown to preferentially induce histone eviction in genomic regions characterized by specific epigenetic modifications [13]. Therefore, three of the agents in our regimen act through epigenetic mechanisms. The results were consistent with our expectations, suggesting that combining epigenetic modifiers with cytotoxic agents was a promising direction for r/r AML treatment.

In this study, NGS was performed on 88 patients, which allowed us to identify prognosis-associated gene mutations. For the 12 patients without detectable gene mutations, the ORR was as high as 66.7%. The underlying mechanism is not clear and needs to be clarified in the future. For patients with epigenetic or transcription factor-related gene mutations but without *FLT3-*ITD, defined as panel ET, the ORR was 59.5%, suggesting that patients with mutations in panel ET should be treated with CDCAG. The finding needs further verification by a prospective, large-scale clinical trial in the future. One study reported that *FLT3-*ITDs were an independent prognostic factor associated with lower OS among patients with r/r AML [32]. Consistent with the study, we found in our trial that patients with *FLT3-*ITDs had a lower chance of achieving CR. Recently, several tyrosine kinase inhibitors (TKIs, e.g., sorafenib, midostaurin, quizartinib, and crenolanib) have been introduced for the treatment of patients with r/r AML with *FLT3* mutations (predominantly ITD). This suggests that TKIs may be a suitable addition to our regimen to treat patients with *FLT3-*ITDs.

Our study was limited in that it followed a traditional single-arm design and did not include a control group. Furthermore, the role of each single agent in this regimen could not be determined.

In conclusion, the CDCAG regimen showed good antileukemic activity and acceptable toxicity. Furthermore, deep sequencing analysis demonstrated that patients with epigenetic or transcription factor-related gene mutations, but without *FLT3-*ITDs, achieved a better response to this combination chemotherapy. Chemotherapy combining epigenetic modifiers with cytotoxic agents may represent a promising direction for patients with r/r AML.

## Methods

### Patients and study design

This single-arm, multicenter, prospective clinical trial evaluated the safety and efficacy of the CDCAG regimen in patients with r/r AML (NCT02886559). This study was conducted in accordance with the Declaration of Helsinki and was approved by the Institutional Review Board at each participating institution. All patients enrolled in the study provided written informed consent.

From June 2016 to June 2018, 93 patients were enrolled at 14 hospitals in China. Adults with r/r AML, defined according to the standard IWG criteria [33], aged between 18 and 60 years, were eligible for this study. Eligible patients must not have received radiotherapy, chemotherapy, targeted therapy, hematopoietic stem cell transplantation, or any other treatment within 4 weeks prior to enrollment. Furthermore, eligible patients were required to have an ECOG performance status [34] ≤ 3 and an expected survival time > 3 months. Patients with active infection, bleeding, new thrombosis, and serious heart, lung, liver, or kidney disease were excluded. The full list of inclusion and exclusion criteria is presented in Table S7.

All patients in this study were treated with the CDCAG regimen (Fig. S4) over a 28-day cycle: chidamide (30 mg, twice per week, days 1–14) and decitabine (20 mg/m^2^/day, days 1–5) in combination with cytarabine (50 mg/m^2^/day, days 1–7 if WBC ≥ 20 × 10^9^/L and days 3–7 if WBC < 20 × 10^9^/L), aclarubicin (10 mg/m^2^/day, days 3–7), and granulocyte colony-stimulating factor (300 μg/day until WBC > 20 × 10^9^/L). Supportive treatment, including anti-infection prophylaxis and growth factor support, was allowed at the investigator’s discretion. If morphologic CRi was achieved [33] and WBC < 2 × 10^9^/L, a subsequent cycle could be delayed by up to 14 days. All patients received two cycles of the CDCAG regimen. Next, hematological response and toxicity were evaluated.

### Definitions of events and end points

Bone marrow aspiration and biopsy were performed upon screening and at day 28 of cycles 1 and 2. Physical exams, clinical laboratory tests, and monitoring of AEs were performed at screening and throughout the study. Response assessments were categorized according to the IWG criteria. The primary end point was treatment success, which was defined as CR or CRi after completion of the CDCAG regimen treatment. The ORR included the CR/CRi rate. AEs were graded according to the National Cancer Institute Common Terminology Criteria for Adverse Events (NCI-CTCAE) version 5.0 (http://ctep.cancer.gov). The definitions of events and end points are described in detail in Table S8.

### Next-generation sequencing and analysis

Bone marrow aspiration samples for mutational analysis were collected before treatment. Genomic DNA extracted from bone marrow was examined for mutations using the target sequencing panel, which covered the entire coding sequences of 127 genes known to be relevant to AML pathogenesis (Annoroad Gene Technology, Table S5). NimbelGen SeqCap EZ Choice was used in accordance with the manufacturer’s protocol, with modifications. Multiplexed libraries were sequenced using 75 bp paired-end runs on Illumina Nextseq 550AR.

Each sample was required to have an average effective depth ≥ 1000× in the target area. Using the Burrows-Wheeler Alignment algorithm to compare the sequence data with the human genome (GRCh37), Picard was used to mark the polymerase chain reaction duplicates, and the quality value of the sequence alignment results was corrected by means of BaseRecalibrator in Genome Analysis Toolkit. MuTect2 software was employed for mutation detection, and all testing mutations were annotated by the ANNOVAR software. The types of analysis included single-nucleotide variants (SNVs), insertions, and deletions (INDELs). Somatic mutations were identified through comparison with the COSMIC (v18) and 1000 Genomes cohort databases, while single-nucleotide polymorphisms described in the dbSNP (v135) database were excluded. The VAF cutoff was set to 0.01 for inclusion in the analyses.

### Statistical analysis

The data cutoff for this report was June 30, 2018. Assessments at screening served as baseline data. Descriptive statistics are presented as the mean ± standard deviations (mean ± SD) for continuous data and as numbers and percentages for dichotomous/categorical data. Chi-square (*χ*^2^)/Fisher’s exact test was used for categorical variables, and the Kruskal-Wallis test was used for continuous variables. Survival functions were estimated using the Kaplan-Meier method and were compared using the log-rank test. Associations with OS were assessed using a Cox proportional hazards model. Logistic regression was used to examine the associations between these variables and response rates. Variables significant at *P* < 0.10 in univariate analyses were entered into an explorative multivariable model. We also adjusted for features that, when added to this model, changed the matched odds ratio by at least 10%. All analyses were performed using Empower Stats (X&Y Solutions, Inc., Boston, MA, USA) and R (version 3.3.3). A two-sided *P* value < 0.05 was considered statistically significant.

### Role of the funding source

Chinese PLA General Hospital investigators designed the study and performed all data collection, data analysis, data interpretation, and wrote the manuscript. The funder National Natural Science Foundation of China had no role in study design, data collection, data analysis, data interpretation, or writing of the report. All authors had full access to all the data in the study and the corresponding author had final responsibility for the decision to submit for publication.

## Supplementary information

**Additional file 1: Table S1.** Gene mutations of patients with r/r AML (N = 88^*^). **Table S2.** Baseline characteristics for patients undergoing gene mutation detection (N = 88). **Table S3.** Clinical responses of patients with different mutations. **Table S4.** Univariate models of overall response rate in patients with different mutations. **Table S5.** Next-generation sequencing of 127-gene mutation panel in r/r AML (Annoroad Gene Technology). **Table S6.** Baseline characteristics and outcomes for patients with detectable mutations (N = 76). **Table S7.** Study inclusion and exclusion criteria. **Table S8.** Definitions of events and end points [1]. **Fig. S1** Overall survival curves for 43 patients who achieved CR/CRi. Data are categorized according to whether the patient underwent allo-HSCT. Abbreviations: allo-HSCT, allogeneic hematopoietic stem cell transplantation. **Fig. S2** The overall response rate of patients with indicated mutations. Abbreviations: CR, complete remission; CRi, complete remission with incomplete blood count recovery; CEBPA-dm, CEBPA double mutation. **Fig. S3** Percentage of patients who achieved and did not achieve CR/CRi among the 88 patients who underwent gene mutation detection. Abbreviations: CR, complete remission; CRi, complete remission with incomplete blood count recovery; CEBPA-dm, CEBPA double mutation. **Fig. S4** Study design. Abbreviations: WBC, white blood cell count; G-CSF, Granulocyte colony-stimulating factor.

## Data Availability

The data that support the findings of this study are available from the Chinese PLA General Hospital, but restrictions apply to the availability of these data, which were used under license for the current study, and, thus, are not publicly available. However, data are available from the authors upon reasonable request and with permission of the Chinese PLA General Hospital.
