# Supplementary material for: Chidamide, decitabine, cytarabine, aclarubicin, and granulocyte colony-stimulating factor (CDCAG) in patients with relapsed/refractory acute myeloid leukemia: a single-arm, phase 1/2 study
Source: Clin Epigenetics. 2020 Sep 1;12:132. doi: 10.1186/s13148-020-00923-4 (PMC7466805; doi:10.1186/s13148-020-00923-4)
Supplement: Supplementary file 1 — Additional file 1: Table S1. Gene mutations of patients with r/r AML (N = 88*). Table S2. Baseline characteristics for patients undergoing gene mutation detection (N = 88). Table S3. Clinical responses of patients with different mutations. Table S4. Univariate models of overall response rate in patients with different mutations. Table S5. Next-generation sequencing of 127-gene mutation panel in r/r AML (Annoroad Gene Technology). Table S6. Baseline characteristics and outcomes for patients with detectable mutations (N = 76). Table S7. Study inclusion and exclusion criteria. Table S8. Definitions of events and end points [1]. Fig. S1 Overall survival curves for 43 patients who achieved CR/CRi. Data are categorized according to whether the patient underwent allo-HSCT. Abbreviations: allo-HSCT, allogeneic hematopoietic stem cell transplantation. Fig. S2 The overall response rate of patients with indicated mutations. Abbreviations: CR, complete remission; CRi, complete remission with incomplete blood count recovery; CEBPA-dm, CEBPA double mutation. Fig. S3 Percentage of patients who achieved and did not achieve CR/CRi among the 88 patients who underwent gene mutation detection. Abbreviations: CR, complete remission; CRi, complete remission with incomplete blood count recovery; CEBPA-dm, CEBPA double mutation. Fig. S4 Study design. Abbreviations: WBC, white blood cell count; G-CSF, Granulocyte colony-stimulating factor. [file 13148_2020_923_MOESM1_ESM.docx]

**Table S1.** Gene mutations of patients with r/r AML (N = 88^*^)

| Gene mutations | Number of mutated genes (%) | Number of mutations |
| --- | --- | --- |
| CEBPA | 17 (19.3) | 27 |
| FLT3-ITD | 17 (19.3) | 17 |
| WT1 | 16 (18.2) | 21 |
| RUNX1 | 14 (15.9) | 14 |
| DNMT3A | 12 (13.6) | 14 |
| NPM1 | 9 (10.2) | 9 |
| GATA2 | 9 (10.2) | 9 |
| TET2 | 8 (9.1) | 10 |
| TP53 | 8 (9.1) | 8 |
| NRAS | 7 (8.0) | 8 |
| KIT | 7 (8.0) | 7 |
| RAD21 | 6 (6.8) | 6 |
| IDH1 | 5 (5.7) | 5 |
| IDH2 | 5 (5.7) | 5 |
| U2AF1 | 4 (4.5) | 5 |
| KRAS | 4 (4.5) | 5 |
| BCOR | 4 (4.5) | 4 |
| PHF6 | 4 (4.5) | 4 |
| EZH2 | 4 (4.5) | 4 |
| ASXL1 | 2 (2.3) | 4 |
| FLT3 | 2 (2.3) | 3 |
| SRSF2 | 2 (2.3) | 2 |
| CSF3R | 2 (2.3) | 2 |
| JAK2 | 2 (2.3) | 2 |
| CALR | 2 (2.3) | 2 |
| KDM6A | 2 (2.3) | 2 |
| PTPN11 | 1 (1.1) | 1 |
| TPMT | 1 (1.1) | 1 |
| CYP3A4 | 1 (1.1) | 1 |
| STAG2 | 1 (1.1) | 1 |
| SH2B3 | 1 (1.1) | 1 |
| IKZF1 | 1 (1.1) | 1 |
| CBL | 1 (1.1) | 1 |
| CREBBP | 1 (1.1) | 1 |
| SMC1A | 1 (1.1) | 1 |
| PDGFRA | 1 (1.1) | 1 |

Abbreviations: CEBPA-dm, CEPBA double mutation.

^*^ Measured in 88 patients who underwent gene mutation detection.

**Table S2.** Baseline characteristics for patients undergoing gene mutation detection (N = 88)

| Characteristic | Without detectable mutation  (n = 12) | With detectable mutations  (n = 76) | *P*-value |
| --- | --- | --- | --- |
| Age, years | 33.8 ± 11.3 | 40.4 ± 12.5 | 0.088 |
| Sex, no. (%) |  |  | 0.842 |
| Male | 7 (58.3) | 42 (55.3%) |  |
| Female | 5 (41.7) | 34 (44.7%) |  |
| BM blasts, % | 0.5 ± 0.3 | 0.4 ± 0.3 | 0.708 |
| HB, g/dL | 87.5 ± 20.8 | 92.5 ± 25.8 | 0.525 |
| WBC, × 10^9^L | 8.5 ± 10.7 | 9.5 ± 17.0 | 0.850 |
| PLT, × 10^9^L | 70.5 ± 66.4 | 69.1 ± 68.3 | 0.947 |
| ECOG PS, no. (%) |  |  | 0.718 |
| 0 | 3 (25.0) | 26 (38.8%) |  |
| 1 | 7 (58.3) | 28 (41.8%) |  |
| 2 | 2 (16.7) | 12 (17.9%) |  |
| 3 |  | 1 (1.5%) |  |
| Diagnosis, no. (%) |  |  | 0.392 |
| Refractory | 6 (50.0) | 27 (35.5%) |  |
| Early relapse | 3 (25.0) | 35 (46.1%) |  |
| Late relapse | 3 (25.0) | 14 (18.4%) |  |
| Prior therapies, no. (%) |  |  | 0.897 |
| 0–5 | 7 (58.3) | 41 (53.9%) |  |
| 6–10 | 3 (25.0) | 24 (31.6%) |  |
| ≥11 | 2 (16.7) | 11 (14.5%) |  |
| Karyotype, no. (%) |  |  | 0.786 |
| Normal karyotype | 6 (50.0%) | 45 (59.2%) |  |
| Complex karyotype^*^ | 1 (8.3%) | 7 (9.2%) |  |
| Others | 5 (41.7%) | 24 (31.6%) |  |

Abbreviations: BM, bone marrow; HB, hemoglobin; WBC, white blood cell count; PLT, platelets; ECOG PS, Eastern Cooperative Oncology Group performance score; CEBPA-dm, CEPBA double mutation.

^*^ Complex karyotype was defined as ≥ 3 clonal chromosomal abnormalities.

**Table S3.** Clinical responses of patients with different mutations

| Mutation^*^ | Functional category | N | Clinical response | | | | |  | CR/CRi | |
| --- | --- | --- | --- | --- | --- | --- | --- | --- | --- | --- |
|  |  |  | CR  N (%) | CRi  N (%) | PR  N (%) | NR  N (%) | ED  N (%) |  | N (%) | *P*-value |
| IDH2 | DNA methylation | 5 | 2 (40.0) | 2 (40.0) | 0 (0.0) | 1 (20.0) | 0 (0.0) |  | 4 (80.0) | 0.088 |
| TET2 | DNA methylation | 8 | 4 (50.0) | 2 (25.0) | 1 (12.5) | 1 (12.5) | 0 (0.0) |  | 6 (75.0) | 0.057 |
| GATA2 | Transcription factor | 9 | 2 (22.2) | 4 (44.4) | 0 (0.0) | 2 (22.2) | 1 (11.1) |  | 6 (66.7) | 0.134 |
| RAD21 | Adhesion and cohesion complex | 6 | 2 (33.3) | 2 (33.3) | 1 (16.7) | 1 (16.7) | 0 (0.0) |  | 4 (66.7) | 0.231 |
| DNMT3A | DNA methylation | 12 | 4 (33.3) | 3 (25.0) | 4 (33.3) | 1 (8.3) | 0 (0.0) |  | 7 (58.3) | 0.256 |
| KIT | Activated signalling | 7 | 3 (42.9) | 1 (14.3) | 1 (14.3) | 2 (28.6) | 0 (0.0) |  | 4 (57.1) | 0.442 |
| WT1 | Transcription factor | 16 | 3 (18.8) | 5 (31.3) | 0 (0.0) | 6 (37.5) | 2 (12.5) |  | 8 (50.0) | 0.550 |
| NPM1 | NPM1 | 9 | 2 (22.2) | 2 (22.2) | 0 (0.0) | 5 (55.6) | 0 (0.0) |  | 4 (44.4) | 0.947 |
| CEBPA-dm | Transcription factor | 9 | 2 (22.2) | 2 (22.2) | 0 (0.0) | 4 (44.4) | 1 (11.1) |  | 4 (44.4) | 0.947 |
| RUNX1 | Transcription factor | 14 | 4 (28.6) | 2 (14.3) | 2 (14.3) | 6 (42.9) | 0 (0.0) |  | 6 (42.9) | 0.962 |
| NRAS | Activated signaling | 7 | 2 (28.6) | 1 (14.3) | 1 (14.3) | 3 (42.9) | 0 (0.0) |  | 3 (42.9) | 0.975 |
| IDH1 | DNA methylation | 5 | 1 (20.0) | 1 (20.0) | 1 (20.0) | 1 (20.0) | 1 (20.0) |  | 2 (40.0) | 0.873 |
| TP53 | Tumor suppressor | 8 | 1 (12.5) | 2 (25.0) | 1 (12.5) | 4 (50.0) | 0 (0.0) |  | 3 (37.5) | 0.721 |
| FLT3-ITD | Activated signaling | 17 | 3 (17.6) | 2 (11.8) | 1 (5.9) | 9 (52.9) | 2 (11.8) |  | 5 (29.4) | 0.186 |

Abbreviations: CR, complete remission; CRi, complete remission with incomplete blood count recovery; PR, partial remission; NR, no response; ED, early death; CEBPA-dm, CEBPA double mutation.

^*^ Mutations detected in more than 5% of patients

**Table S4.** Univariate models of overall response rate in patients with different mutations

| Mutation^*^ | N (%) | OR (95% CI) | *P*-value |
| --- | --- | --- | --- |
| CEBPA |  |  |  |
| No | 59 (77.63%) | 1.0 |  |
| Yes | 17 (22.37%) | 0.89 (0.30–2.65) | 0.8322 |
| FLT3-ITD |  |  |  |
| No | 59 (77.63%) | 1.0 |  |
| Yes | 17 (22.37%) | 0.46 (0.14–1.47) | 0.1918 |
| WT1 |  |  |  |
| No | 60 (78.95%) | 1.0 |  |
| Yes | 16 (21.05%) | 1.40 (0.46–4.23) | 0.5511 |
| RUNX1 |  |  |  |
| No | 62 (81.58%) | 1.0 |  |
| Yes | 14 (18.42%) | 0.97 (0.30–3.14) | 0.9624 |
| DNMT3A |  |  |  |
| No | 64 (84.21%) | 1.0 |  |
| Yes | 12 (15.79%) | 2.05 (0.59–7.15) | 0.2621 |
| NPM1 |  |  |  |
| No | 67 (88.16%) | 1.0 |  |
| Yes | 9 (11.84%) | 1.05 (0.26–4.25) | 0.9474 |
| CEBPA-dm |  |  |  |
| No | 67 (88.16%) | 1.0 |  |
| Yes | 9 (11.84%) | 1.05 (0.26–4.25) | 0.9474 |
| GATA2 |  |  |  |
| No | 67 (88.16%) | 1.0 |  |
| Yes | 9 (11.84%) | 2.96 (0.68–12.88) | 0.1474 |
| TET2 |  |  |  |
| No | 68 (89.47%) | 1.0 |  |
| Yes | 8 (10.53%) | 4.56 (0.86–24.26) | 0.0756 |
| TP53 |  |  |  |
| No | 68 (89.47%) | 1.0 |  |
| Yes | 8 (10.53%) | 0.76 (0.17–3.44) | 0.7216 |
| NRAS |  |  |  |
| No | 69 (90.79%) | 1.0 |  |
| Yes | 7 (9.21%) | 0.98 (0.20–4.69) | 0.9748 |
| KIT |  |  |  |
| No | 69 (90.79%) | 1.0 |  |
| Yes | 7 (9.21%) | 1.84 (0.38–8.85) | 0.4473 |
| RAD21 |  |  |  |
| No | 70 (92.11%) | 1.0 |  |
| Yes | 6 (7.89%) | 2.83 (0.49–16.48) | 0.2478 |
| IDH1 |  |  |  |
| No | 71 (93.42%) | 1.0 |  |
| Yes | 5 (6.58%) | 0.86 (0.14–5.47) | 0.8732 |
| IDH2 |  |  |  |
| No | 71 (93.42%) | 1.0 |  |
| Yes | 5 (6.58%) | 5.79 (0.62–54.52) | 0.1246 |
| COMUT_FLT3-ITD&NPM1 |  |  |  |
| No | 81 (92.05%) | 1.0 |  |
| Yes | 7 (7.95%) | 0.43 (0.08, 2.35) | 0.3306 |
| COMUT_ FLT3-ITD&DNMT3A |  |  |  |
| No | 82 (93.18%) | 1.0 |  |
| Yes | 6 (6.82%) | 1.16 (0.22, 6.08) | 0.8624 |
| COMUT_NPM1&DNMT3A |  |  |  |
| No | 83 (94.32%) | 1.0 |  |
| Yes | 5 (5.68%) | 1.78 (0.28, 11.19) | 0.5406 |
| COMUT_WT1&CEBPA-dm |  |  |  |
| No | 83 (94.32%) | 1.0 |  |
| Yes | 5 (5.68%) | 0.75 (0.12, 4.74) | 0.7616 |
| COMUT_FLT3-ITD&WT1 |  |  |  |
| No | 84 (95.45%) | 1.0 |  |
| Yes | 4 (4.55%) | 0.37 (0.04, 3.67) | 0.3932 |
| COMUT_FLT&RUNX1 |  |  |  |
| No | 84 (95.45%) | 1.0 |  |
| Yes | 4 (4.55%) | 0.37 (0.04, 3.67) | 0.3932 |
| COMUT_CEBPA-dm&GATA2 |  |  |  |
| No | 84 (95.45%) | 1.0 |  |
| Yes | 4 (4.55%) | 1.15 (0.16, 8.58) | 0.8888 |

^*^ Mutations detected in more than 5% of patients

**Table S5.** Next-generation sequencing of 127-gene mutation panel in r/r AML (Annoroad Gene Technology)

| Functional category^*^ | | Gene mutations | N (%) |
| --- | --- | --- | --- |
| Epigenetic modifier | DNA methylation | DNMT3A, TET2, IDH1, IDH2, TPMT, MLL | 6 (6.8) |
|  | Histone methylation | KDM6A, EZH2, SETD2, KMT2C | 4 (4.5) |
|  | Histone acetylation | CREBBP, EP300 | 2 (2.3) |
| Transcription factor | | WT1, CEBPA, RUNX1, GATA1, GATA2, GATA3, ETV6, IKZF1, CUX1, ERG, MEF2B, RB1, STAT5B, STAT5A, TCF3, GFI1 | 16 (18.2) |
| Other chromatin modifiers | | ASXL1, BCORL1, BCOR, ID3, SETBP1, PHF6, BLM | 7 (8.0) |
| NPM1 | | NPM1 | 1 (1.1) |
| Tumor suppressor | | TP53, MPL, APC, CBLB, CBLC, MDM2, CDKN2A | 7 (8.0) |
| Activated signaling | | FLT3, CBL, NRAS, KRAS, HRAS, PTPN11, KIT, CSF1R, CSF3R, JAK1, JAK2, JAK3, SH2B3, NTRK1, NTRK2, ABL, CRLF2, AKT2, AKT3, AMER1, ATM, ATRX, BRAF, EGFR, IL7R, MAP2K4, MAP3K7, NOTCH1, NOTCH2, PIK3CA, PTEN, PDGFRA, SMAD4, SYK, TRAF3, NF1, ETNK1, STAT3, MYD88 | 39 (44.3) |
| Spliceosome and RNA metabolism | | SF3B1, SRSF2, U2AF1, ZRSR2, PRPF8, DDX41 | 6 (6.8) |
| Cell metabolism | | GSTP1, NT5C2, NQO1, PIGA, MTHFR, ELA2, CYP2C19, CYP3A4, GSTM1 | 9 (10.2) |
| Adhesion and cohesion complex | | RAD21, STAG2, SMC1A, SMC3, FAT1 | 5 (5.7) |
| Others | | ABCB1, ABCC3, BCL2, CACNA1E, CARD11, CCND1, CD79B, CDA, DIS3, ERCC1, CTLA4, FAM46C, FBXW7, GNAS, MLH1, NF2, TERC, XRCC1, CALR, BIRC3, SRP72, HAX1, TERT, DNAH9, DKC1 | 25 (28.4) |

^*^Mutations were categorized according to their function[^1-4^](#_ENREF_1).

**Table S6.** Baseline characteristics and outcomes for patients with detectable mutations (N = 76)

| Characteristic | Other mutations  (n = 39) | Mutations in panel ET^*^  (n = 37) | *P*-value |
| --- | --- | --- | --- |
| CR/CRi, no. (%) |  |  | 0.006 |
| No | 28 (71.8) | 15 (40.5) |  |
| Yes | 11 (28.2) | 22 (59.5) |  |
| Age, years | 41.0 ± 12.6 | 39.7 ± 12.5 | 0.647 |
| Sex, no. (%) |  |  | 0.474 |
| Male | 20 (51.3) | 22 (59.5) |  |
| Female | 19 (48.7) | 15 (40.5) |  |
| BM blasts, % | 0.5 ± 0.3 | 0.4 ± 0.3 | 0.363 |
| HB, g/dL | 92.3 ± 22.2 | 92.6 ± 29.3 | 0.959 |
| WBC, × 10^9^L | 11.8 ± 19.6 | 7.0 ± 13.6 | 0.223 |
| PLT, × 10^9^L | 65.9 ± 57.3 | 72.5 ± 79.0 | 0.679 |
| ECOG PS, no. (%) |  |  | 0.518 |
| 0 | 14 (38.9) | 12 (38.7) |  |
| 1 | 13 (36.1) | 15 (48.4) |  |
| 2 | 8 (22.2) | 4 (12.9) |  |
| 3 | 1 (2.8) | 0 (0.0) |  |
| Diagnosis, no. (%) |  |  | 0.857 |
| Refractory | 15 (38.5) | 12 (32.4) |  |
| Early relapse | 17 (43.6) | 18 (48.6) |  |
| Late relapse | 7 (17.9) | 7 (18.9) |  |
| Prior therapies, no. (%) |  |  | 0.255 |
| 0–5 | 24 (61.5) | 17 (45.9) |  |
| 6–10 | 9 (23.1) | 15 (40.5) |  |
| ≥11 | 6 (15.4) | 5 (13.5) |  |
| Karyotype, no. (%) |  |  | 0.870 |
| Normal karyotype | 22 (56.4%) | 23 (62.2%) |  |
| Complex karyotype^#^ | 4 (10.3%) | 3 (8.1%) |  |
| Others | 13 (33.3%) | 11 (29.7%) |  |

Abbreviations: CR, complete remission; CRi, complete remission with incomplete blood count recovery; BM, bone marrow; HB, hemoglobin; WBC, white blood cell count; PLT, platelets; ECOG PS, Eastern Cooperative Oncology Group performance score.

^*^ Panel ET: Epigenetic modifier-related or transcription factor-related gene mutations, but without FLT3-ITD co-mutation.

^#^ Complex karyotype was defined as ≥ 3 clonal chromosomal abnormalities.

**Table S7.** Study inclusion and exclusion criteria

| Inclusion criteria |  |
| --- | --- |
|  | Adults aged ≥ 18 and ≤ 60 years |
|  | Patients diagnosed with AML according to the 2008 WHO myeloid malignant disease diagnosis standard |
|  | Patients who relapsed after remission or who did not achieve remission after at least two cycle of systemic therapy (including chemotherapy, hematopoietic stem cell transplantation, and others) |
|  | ECOG performance status 0−3 |
|  | Expected survival time ˃ 3 months |
|  | Patients without serious heart, lung, liver, or kidney disease |
|  | Patients who have not received radiotherapy, chemotherapy, targeted therapy, hematopoietic stem cell transplantation, or any other treatment within 4 weeks prior to enrolment |
|  | Patients able to understand and willing to sign informed consent |
| Exclusion criteria |  |
|  | Patients who are allergic to the study drug or drugs with similar chemical structures |
|  | Bone marrow hyperplasia and WBC < 2.0×109/L |
|  | Pregnant or lactating women, and women of childbearing age who do not want to practice effective methods of contraception |
|  | Active infection |
|  | Active bleeding |
|  | Patients with new thrombosis, embolism, cerebral hemorrhage, or other diseases or medical history within one year prior to enrolment |
|  | Patients with mental disorders or other conditions whereby informed consent cannot be obtained, and where the requirements of the study treatment and procedures cannot be met |
|  | Cardiac ultrasound showing a diastolic pericardial fluid dark area with a width ˃ 10 mm |
|  | Patients with a history of clinically significant QTc interval prolongation (male > 450 ms; female > 470 ms), ventricular heart tachycardia and atrial fibrillation, II-degree heart block, myocardial infarction attack within one year prior to the enrolment, congestive heart failure, and patients with coronary heart disease who have clinical symptoms and need drug treatment |
|  | Liver function abnormalities (total bilirubin > 1.5 times the upper limit of the normal range, ALT/AST > 2.5 times the upper limit of the normal range or patients with liver involvement whose ALT/AST > 1.5 times the upper limit of the normal range), renal anomalies (serum creatinine > 1.5 times the upper limit of the normal value) |
|  | Surgery on the main organs within the past six weeks |
|  | Drug abuse or long-term alcohol abuse that would affect the evaluation results |
|  | Patients who have received organ transplants |
|  | Patients not suitable for the study according to investigator's assessment |

Abbreviations: WHO, World Health Organization; ECOG, Eastern Cooperative Oncology Group; WBC, white blood cell count; QTc, corrected QT interval; ALT, glutamic-pyruvic transaminase; AST, glutamic oxaloacetic transaminase.

**Table S8.** Definitions of events and end points [1]

| Events and end points | Abbreviation | Definition |
| --- | --- | --- |
| Primary refractory |  | Failure to achieve CR1 after one or two cycles of induction |
| Relapse |  | Reappearance of leukemic blasts in the peripheral blood, or the finding of more than 5% blasts in the bone marrow, or extramedullary relapse |
| Early relapse |  | Relapse within one year from start of treatment |
| Late relapse |  | Relapse after one year from start of treatment |
| Early death |  | Death within 28 days from start of treatment |
| Complete response | CR | Bone marrow shows normal hematopoiesis, bone marrow blasts < 5%, no blasts with Auer rods or persistence of extramedullary disease, absolute neutrophil count > 1 × 10^9^/L, platelets ≥ 100 × 10^9^/L, no residual evidence of extramedullary disease |
| CR with incomplete blood  count recovery | CRi | All CR criteria except for residual neutropenia (< 1.0 × 10^9^/L) or thrombocytopenia (< 100 × 10^9^/L) |
| Overall survival | OS | Measured from the date of diagnosis to the date of last follow-up or death |
| Relapse-free survival | RFS | Measured from the date of attaining CR1 until the first relapse, death, or the final follow-up day |

Abbreviations: CR1, first complete remission.

**Fig. S1** Overall survival curves for 43 patients who achieved CR/CRi. Data are categorized according to whether the patient underwent allo-HSCT.


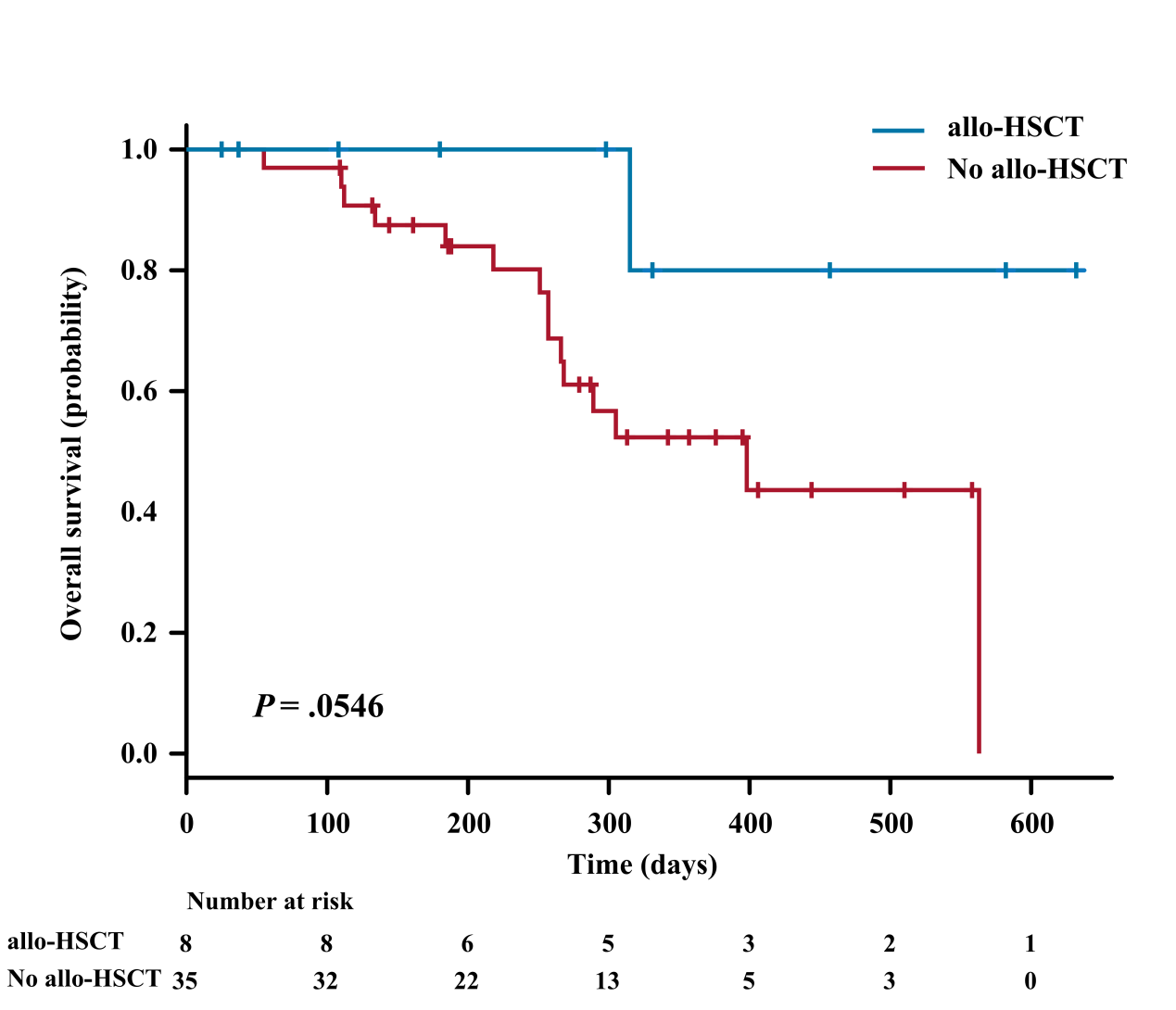


Abbreviations: allo-HSCT, allogeneic hematopoietic stem cell transplantation

**Fig. S2** The overall response rate of patients with indicated mutations.

Abbreviations: CR, complete remission; CRi, complete remission with incomplete blood count recovery; CEBPA-dm, CEBPA double mutation.**Fig. S3** Percentage of patients who achieved and did not achieve CR/CRi among the 88 patients who underwent gene mutation detection.

Abbreviations: CR, complete remission; CRi, complete remission with incomplete blood count recovery; CEBPA-dm, CEBPA double mutation.

**Fig. S4** Study design.


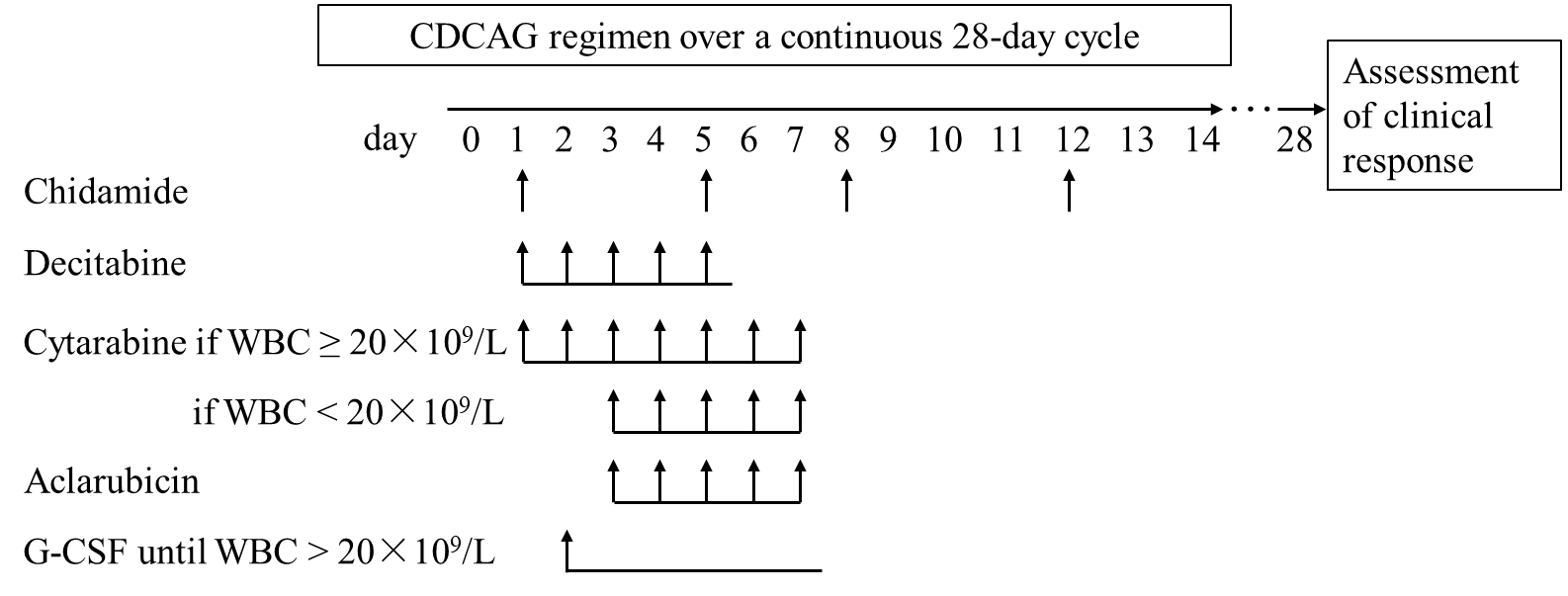


Abbreviations: WBC, white blood cell count; G-CSF, Granulocyte colony-stimulating factor.

**References**

1. Cheson BD, Bennett JM, Kopecky KJ, Buchner T, Willman CL, Estey EH *et al*. Revised recommendations of the International Working Group for Diagnosis, Standardization of Response Criteria, Treatment Outcomes, and Reporting Standards for Therapeutic Trials in Acute Myeloid Leukemia. J Clin Oncol. 2003; 21: 4642-4649.
